# Fast food diet-induced non-alcoholic fatty liver disease exerts early protective effect against acetaminophen intoxication in mice

**DOI:** 10.1186/s12876-017-0680-z

**Published:** 2017-11-28

**Authors:** Tae Hyung Kim, Dahee Choi, Joo Young Kim, Jeong Hyeon Lee, Seung-Hoi Koo

**Affiliations:** 1 Department of Internal Medicine, Korea University College of Medicine, Seoul, South Korea; 2 Division of Life Sciences, Korea University College of Life Sciences & Biotechnology, 145 Anam-Ro Seongbuk-Gu, Seoul, 02841 South Korea; 3 Department of Pathology, Korea University College of Medicine, Seoul, South Korea

**Keywords:** Acetaminophen, Non-alcoholic fatty liver disease, Drug-induced liver injury, Peroxisome proliferator-activated receptor gamma

## Abstract

**Background:**

Acetaminophen (APAP) is a readily available and safe painkiller. However, its overdose is the most common cause of acute liver injury (ALI). Many predisposing factors contribute to susceptibility to APAP-induced ALI. Non-alcoholic fatty liver disease (NAFLD), the major cause of chronic liver disease, is considered an important predictor of APAP-induced ALI, although the exact mechanism controversial. In this study, we aimed to elucidate the effects of NAFLD on APAP-induced ALI.

**Methods:**

Two groups of mice, normal chow (NC) diet-fed and fast food (FF) diet-fed mice for 14 weeks, were further divided into two subgroups: intraperitoneally injected with either saline (NC-S and FF-S groups) or APAP (NC-A and FF-A groups). Biochemical tests, histological analysis, quantitative PCR, and western blotting were conducted.

**Results:**

Alanine aminotransferase (ALT) level (199.0 ± 39.0 vs. 63.8 ± 7.4 IU/L, *p* < 0.05) and NAFLD activity score (0 vs. 4.5 ± 0.22) were significantly higher in mice in FF-S group than those in NC-S group. ALI features such as ALT level (8447.8 ± 1185.3 vs. 836.6 ± 185.1 IU/L, *p* < 0.001) and centrizonal necrosis were prominent and mRNA levels of *Trib3* (RR, 1.81) was high in mice in the NC-A group. Levels of CYP2E1 and anti-inflammatory molecules such as PPAR-γ, p62, and NRF2 were high in mice in the FF-A group.

**Conclusions:**

Our results showed that while the FF diet clearly induced non-alcoholic steatohepatitis and metabolic syndrome, NAFLD also attenuates APAP-induced ALI by inducing anti-inflammatory molecules such as PPAR-γ.

**Electronic supplementary material:**

The online version of this article (10.1186/s12876-017-0680-z) contains supplementary material, which is available to authorized users.

## Background

Acetaminophen (APAP) is a readily available and commonly used antipyretic and analgesic drug worldwide [1–3]. It is safe when administered within its therapeutic dose; however, its overdose is the most common (50%) cause of acute liver failure in the US and other Western countries [2, 3]. APAP-induced acute liver injury (ALI) is a dose-dependent hepatocellular injury. APAP is metabolized by P450 enzymes, predominantly cytochrome P450 2E1 (CYP2E1), and is converted to *N*-acetyl-*p*-benzoquinone imine (NAPQI). NAPQI is detoxified by glutathione (GSH), and its accumulation results in GSH depletion [4]. In addition, this event is followed by mitochondrial dysfunction, reactive oxygen species (ROS) generation, and necrotic cell death. Pathologically, APAP-induced ALI is characterized by confluent centrizonal necrosis [5]. Treatment of this usually involves early *N*-acetylcysteine therapy and supportive care; however, emergent liver transplantation is required in some cases [6].

Non-alcoholic fatty liver disease (NAFLD) is considered as a risk factor for APAP-induced ALI [7]. NAFLD affects up to 20–40% of the population worldwide [8, 9] and is a major cause of liver-related morbidity and mortality [10]. Non-alcoholic steatohepatitis (NASH), severe form of NAFLD, accompanies with oxidative stress, which were also considered to lead severe APAP-induced ALI.

However, in vivo experiments have provided inconsistent results. Majority of studies indicate that rodents with NAFLD develop severe APAP-induced ALI [11–13]. Conversely, some studies have shown that rodents with NAFLD are resistant to APAP-induced ALI [14, 15]. This discrepancy may be due to the differences in rodent species, APAP dose administered, administration method, and the establishment of NAFLD.

Many methods such as genetic modification [11, 12] and different diets [13, 14, 16] have been used to establish animal models of NAFLD. The occurrence of NAFLD in humans has been increased because of increased consumption of fast food (FF) and fructose containing beverage consumption [9]. Rodent models of FF diet-induced NAFLD have been established using diets that mimic human FF diet; moreover, these models are different from previously established animal models of NAFLD [17, 18]. However, an animal model of FF-induced NAFLD has not yet been used for assessing APAP-induced ALI.

The present study elucidated the early effects of NAFLD on APAP-induced ALI and determined underlying mechanisms. To our knowledge, this is the first APAP-related study involving a mouse model of FF diet-induced NAFLD.

## Methods

### Animal treatment

Twenty-eight C57BL/6 mice with age of eight-week and male were purchased from Orient Bio, Inc., (Seongnam, Korea). The mice were randomly assigned to two dietary groups, i.e., mice fed normal chow (NC) diet (#1314 Altromin Diet, Lage, Germany) and mice fed FF diet. Mice in the FF diet group received a diet that was relatively rich in saturated fats, cholesterol, and fructose and provided 41% energy from fat (milk fat, 12% saturated), with 0.2% cholesterol (D12079B, Western diet, Research Diet, New Brunswick, NJ). In addition, FF diet-fed mice received high-fructose corn syrup mixed in the drinking water. The details of both the diets are given in Additional file 1: Table S1. All the mice had free access to food and water and were fed their respective diets for 14 weeks. APAP (A7085) were purchased from Sigma-Aldrich (St. Louis, MO), was dissolved in warm saline, and intraperitoneally injected into mice at a dose of 200 mg/kg body weight. Control mice in each group were injected with saline. All the mice were sacrificed by performing cervical dislocation, and their livers were removed immediately. Total number of 28 mice were allocated to 4 groups as following: six mice to NC diet with saline injection (NC-S) group and FF diet with saline injection (FF-S) group, and eight mice to NC diet with APAP injection (NC-A) group and FF diet with (FF-A) group. All the procedures were performed in accordance with the guidelines of the Institutional Animal Care and Use Committee of the Korea University. This study was also approved by the same committee (KUIACUC-2016-81).

### Biochemical analysis

Serum alanine aminotransferase (ALT), glucose, and cholesterol levels were assayed using automated clinical chemistry analyzer (DRI-CHEM 4000i; FujiFilm, Tokyo). Serum insulin and liver triglyceride levels were measured using commercial kits (Alpco, Salem, NH and Wako, Osaka, Japan, respectively).

### Western blot analysis

Hepatic expressions of CYP2E1, peroxisome proliferator-activated receptor gamma (PPAR-γ), nuclear factor-kappa B (NF-κB), extracellular signal-regulated kinase (ERK), phosphorylated ERK (p-ERK), phosphorylated map kinase kinase 4 (p-MKK4), phosphorylated c-Jun N-terminal kinases (p-JNK), p62, phosphorylated p62 (p-p62), nuclear factor erythroid 2-related factor 2 (NRF2), β-actin were determined by performing western blotting. Similar-weighted liver sections obtained from mice in each group were homogenized in lysis buffer and centrifuged. Proteins were separated by performing electrophoresis on 4–12% gradient Bis-Tris gels; transferred to Hybond ECL nitrocellulose membranes; and immunoblotted using anti-CYP2E1 (Abcam, Cambridge, UK), anti-PPAR-γ (Cell Signaling, Danvers, MA), anti-NF-κB (Cell Signaling), anti-ERK (Cell Signaling), anti-p-ERK T202/Y204 (Cell Signaling), anti-p-MKK4 S257 (Cell Signaling), anti-p-JNK T183/Y185 (Cell Signaling), anti-p62 (Cell Signaling), anti-p-p62 S349 (Cell Signaling), and anti-NRF2 (Cell Signaling) antibodies. Antibody against β-actin (Sigma-Aldrich) was used to assess equal loading.

### Reverse transcription-quantitative polymerase chain reaction

For performing reverse transcription-quantitative polymerase chain reaction (RT-qPCR), total RNA from the mouse liver was extracted using RNeasy Mini Kit (QIAGEN GmbH, Hilden, Germany). Complementary DNA was synthesized using Superscript II enzyme (Gibco/Invitrogen, Grand Island, NY) and was analyzed by performing qPCR with SYBR Green PCR Kit and TP800 Thermal Cycler Dice Real Time System (Takara Bio Inc., Otsu, Japan). All data were normalized to those obtained for the gene encoding ribosomal L32. All primers used for performing qPCR are listed in Additional file 1: Table S2. Differences in gene expression were considered significant when the relative ratio (RR) of mRNA levels between two groups was more than or equal to 1.5.

### Histological analysis

Similar-sized portions of the left lateral lobe obtained from the liver of mice in each group were fixed in 10% neutral-buffered formalin for 24 h. Next, the liver portions were trimmed, sectioned into approximately 5-μm-thick sections, processed, and stained with hematoxylin and eosin (H&E) [19] and Masson’s trichrome stain. Terminal deoxynucleotidyl transferase-mediated dUTP nick-end labeling (TUNEL) assay was performed by staining the liver sections with In Situ Cell Death Detection Kit, AP (Roche Diagnostics, Indianapolis, IN), according to the manufacturer’s instructions [20]. Histological analysis was performed by two experienced pathologists in a blinded manner. NAFLD severity was evaluated using NAFLD activity score, composed of steatosis, ballooning, and lobular inflammation.

### Statistical analysis

Results are expressed as mean ± standard deviation (SD) or standard error of the mean (SEM), as indicated in figure legends. Statistical differences were assessed by Student’s *t*-test, with *p* < 0.05 being considered statistically significant.

## Results

### Manifestation of NAFLD

**Fig. 1:**
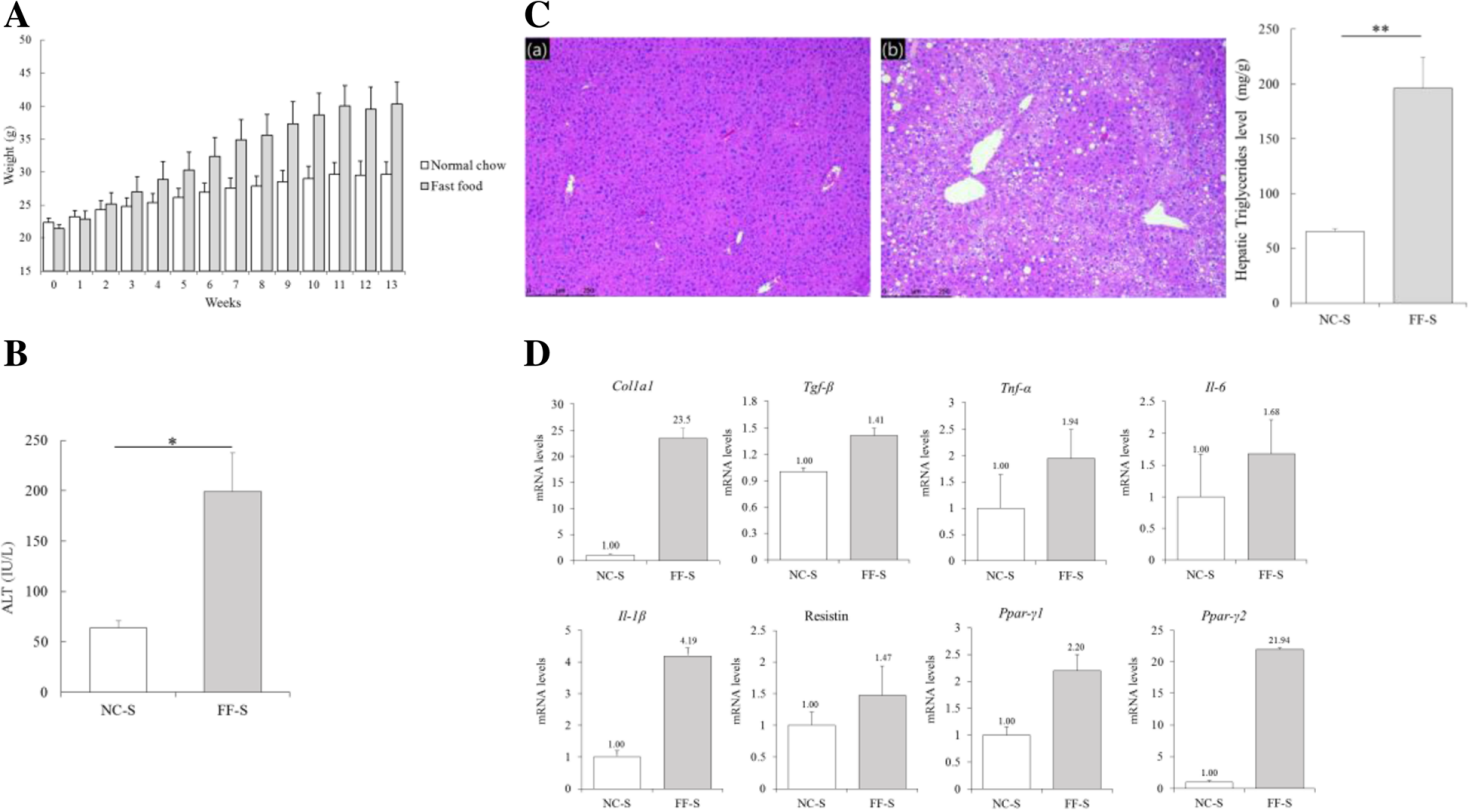
Manifestation of the NAFLD characteristics. (A) Changes in the body weight over time in mice fed the NC and FF diets (12–16 mice per group) are presented as mean ± SEM. (B) Plasma ALT levels at 6 h after saline injection in mice in the NC-S and FF-S groups; each bar represents mean ± SEM value of 6–8 mice per group. (C) H&E-stained liver sections of mice in the (a) NC-S and (b) FF-S groups. (b); magnification, ×200. The total hepatic triglyceride levels were plotted. (D) Relative mRNA levels of genes encoding hepatic markers in mice in the NC-S and FF-S groups. RT-qPCR was performed to determine the mRNA expression. Data are presented as mean ± SD values obtained over measurements carried out thrice by pooling 6–8 mice per group. Relative values were compared with the mean value obtained for mice in the NC-S group. ALT, alanine transaminase. *p < 0.05 and **p < 0.01

**Fig. 2:**
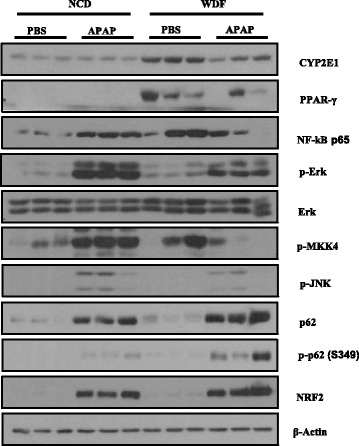
Western blotting analysis of hepatic molecules.Levels of hepatic anti-inflammatory and inflammatory proteins of all groups were showed. β-Actin was used to confirm equal protein loading

Mice fed FF diet for 3 weeks were significantly more obese than those fed NC diet for three weeks (*p* < 0.05). The average weight after overnight fasting was 38.3 and 27.1 g for mice fed FF and NC diets at 14 weeks, respectively (Fig. 1A). Mice in the FF-S group showed higher plasma fasting glucose (*p* = 0.006), cholesterol (*p* = 0.01), and insulin (*p* = 0.014) levels than mice in the NC-S group (Table 1). Moreover, mice in the FF-S group showed significantly higher homeostasis model assessment of insulin resistance (HOMA-IR) index than those in the NC-S group. Mice in the FF-S group also had higher ALT level compared with the counterparts in the NC-S group (199.0 ± 39.0 vs. 63.8 ± 7.4 IU/L, *p* 0.017; Fig. 1B). Histological analysis (Fig. 1C) detected higher degree of hepatic steatosis and lobular inflammation in mice fed FF diet than in mice fed NC diet (*p* < 0.001). NAFLD activity score that integrated the findings of histological analysis was significantly different between mice in the FF-S and NC-S groups (*p* < 0.001; Table 2). The difference of degree of steatosis between FF-S and NC-S groups was more pronounced at the hepatic triglyceride level (196.4 ± 28.3 vs. 65.2 ± 2.8 mg/g, *p* = 0.003; Fig. 1C). Masson’s trichrome-stained liver sections of mice in the FF-S group did not show definite fibrosis (data not shown). However, mRNA levels of genes encoding liver fibrosis markers COL1a1 (RR = 23.5) and TGF-β (RR = 1.41), were higher in mice in the FF-S group than in mice in the NC-S group (Fig. 1D). In addition, mRNA levels of genes encoding proinflammatory markers TNF-α, IL-6, and IL-1β (RR = 1.94, 1.68, and 4.19, respectively) were significantly increased in mice in the FF-S group. Moreover, mRNA levels of genes encoding resistin, a marker of insulin resistance, and PPAR-γ were higher in mice in the FF-S group than in mice in the NC-S group (RR = 1.47, 2.20 for PPAR-γ1 and 21.94 for PPAR-γ2). Western blot analysis (Fig. 2) showed prominent CYP2E1, PPAR-γ, NF-κB, p-ERK, and p-MKK4 expression in the tissue homogenates of mice in the FF-S group than in those of mice in the NC-S group. These findings are characteristics of NAFLD.

**Table 1:** Serum biochemical profiles of mice in the NC-S and FF-S groups

	NC-S	FF-S	*p*-Value
Glucose, mg/dL	129.2 ± 7.4	161.7 ± 2.5	0.006
Serum cholesterol, mg/dL	89.2 ± 2.4	190.5 ± 12.8	0.010
Insulin, mU/L	43.84 ± 11.58	159.83 ± 29.47	0.014
HOMA-IR	14.81 ± 3.40	65.56 ± 12.50	0.013

Results are presented as mean ± SEM

*FF-S* mice fed the fast food diet and injected with saline (*n* = 6), *HOMA-IR* homeostasis model assessment of insulin resistance, *NC-S* mice fed the normal chow diet and injected with saline (*n* = 6)

**Table 2:** Histological analysis of H&E-stained liver sections of mice in each group

	NC-S	FF-S	NC-A	FF-A	*p*-Value
Steatosis (0–3)	0.0 ± 0	2.75 ± 0.10	0.0 ± 0	2.94 ± 0.18	0.181†
Lobular inflammation (0–3)	0.0 ± 0	1.58 ± 0.23	0.0 ± 0	1.67 ± 0.14	0.957†
Hepatocellular ballooning (0–2)	0.0 ± 0	0.17 ± 0.14	0.0 ± 0	0.88 ± 0.13	0.006†
NAS	0.0 ± 0	4.50 ± 0.22	0.0 ± 0	5.38 ± 0.32	0.034†
Centrizonal necrosis (0–3)	0.0 ± 0	0.0 ± 0	1.75 ± 0.30	0.0 ± 0	–

Results are presented as mean ± SEM

The degree of necrosis was expressed as the mean of 10 different fields and has been classified as 0, normal; 1, mild; 2, moderate; 3, severe. †Student’s *t*-test to determine differences between mice in the FF-S and FF-A groups

*FF-A* mice fed the fast food diet and injected with APAP (*n* = 8), *NAS* non-alcoholic fatty liver disease activity score, *NC-A* mice fed the normal chow diet and injected with acetaminophen (*n* = 8)

### Presentation of APAP-induced ALI

**Fig. 3:**
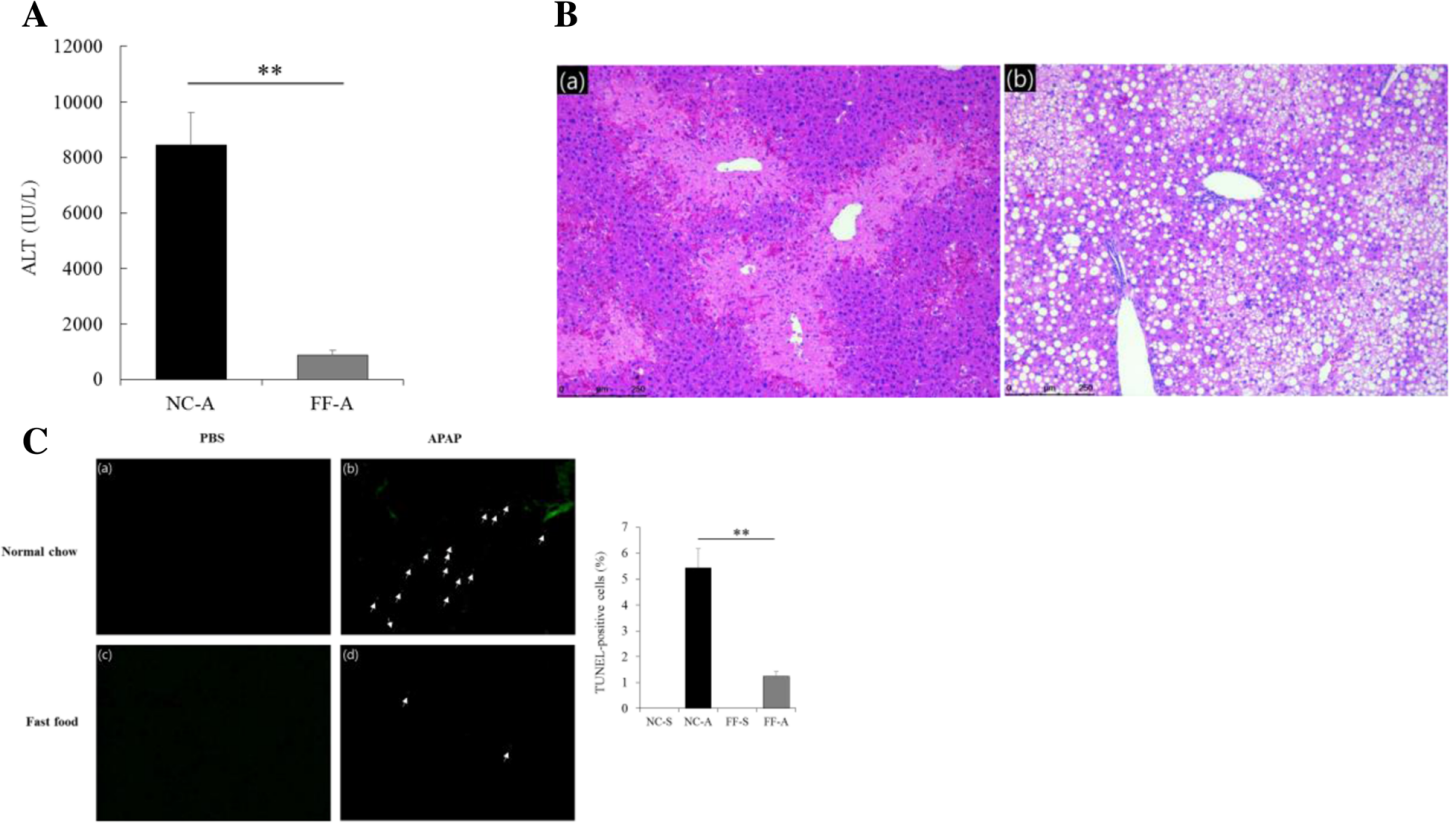
Manifestation of APAP-induced ALI characteristics. (A) Plasma ALT levels in mice in the NC-A and FF-A groups. Plasma ALT level significantly increased in mice in the NC-A group. (B) H&E-stained liver sections: (a) extensive sterile necrosis around the central veins in mice in the NC-A group and (b) steatosis observed in mice in the FF-A group; magnification, ×200. (C) TUNEL-stained liver sections. DNA fragmentation was not observed in the liver sections of mice in the (a) NC-S and (c) FF-S groups. DNA fragmentation was higher in mice in the NC-A group (b) than in mice in the FF-A group (d); magnification, ×200. Average percentages of TUNEL-positive cells in mice in the four groups are plotted. **p < 0.01

ALT levels were higher in mice in the NC-A group than in mice in the FF-A group (8447.8 ± 1185.3 vs. 836.6 ± 185.1 IU/L, *p* < 0.001; Fig. 3A). Histological analysis (Fig. 3B) showed prominent centrizonal necrosis around the central veins in mice in the NC-A group and no necrosis in mice in the FF-A group (Table 2). Results of the TUNEL assay for assessing cell damage (Fig. 3C) showed DNA fragmentation in mice in the NC-A group compared with that in mice in the FF-A group (5.4% vs. 1.3%, *p* = 0.001).

### Levels of enzymes associated with APAP metabolism

**Fig. 4:**
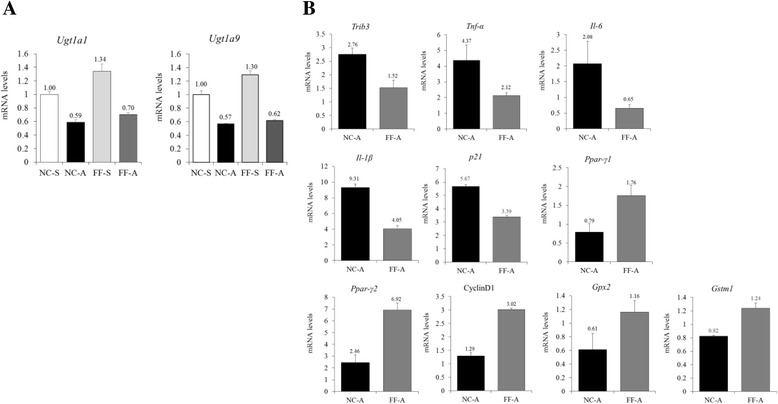
RT-qPCR of hepatic molecules associated with APAP metabolism and oxidative stress. a Relative mRNA levels of genes encoding hepatic UGT1A1 and UGT1A9. These mRNA levels were not significantly different between mice in the NC-S and FF-S groups. **b** Relative mRNA levels of genes encoding hepatic molecules between NC-A and FF-A groups. Lower level of inflammatory enzymes and higher level of antioxidant enzymes are presented in FF-A group

The mRNA levels of the genes encoding uridine diphosphate glucuronosyltransferase 1A1 (UGT1A1) and UGT1A9, which are the key enzymes involved in glucuronidation, were not significantly different between mice in the NC-S and FF-S groups (RR = 1.34 and 1.30; Fig. 4A). Results of western blot analysis showed higher CYP2E1 expression in mice fed FF diet than in mice fed NC diet (Fig. 2).

### Molecules associated with APAP-induced ALI

The mRNA level of Tribbles homolog 3 gene (*Trib3*), which indirectly reflects GSH depletion [21], was higher in mice in the NC-A group than in mice in the FF-A group (RR = 1.82; Fig. 4B). The mRNA levels of genes encoding TNF-α, IL-6, IL-1β, and p21 were higher in mice in the NC-A group than in mice in the FF-A group (RR = 2.06, 3.21, 2.30, and 1.68, respectively). Results of western blot analysis showed higher NF-κB, p-ERK, p-MKK4, and p-JNK expression in tissue homogenates of mice in the NC-A group than in those of mice in the FF-A group (Fig. 2), indicating the increased proinflammatory signals in the liver. Conversely, the mRNA levels of genes encoding PPAR-γ, cyclin-D1, GPX2, and GSTM1 were lower in mice in NC-A group than in mice in the FF-A group (RR = 0.45, 0.43, 0.53, and 0.66, respectively; Fig. 4B). Results of western blot analysis showed higher PPAR-γ, p62, p-p62, and NRF2 expression in tissue homogenates of mice in the FF-A group than in those of mice in the NC-A group (Fig. 2), suggesting that increased anti-oxidant response and anti-inflammatory signals. That may exert a protection effect against APAP.

## Discussion

In the present study, NAFLD was successfully established in mice by administering the FF diet supplemented with fructose for 14 weeks. Biochemical analysis of blood samples showed hypercholesterolemia, high fasting serum glucose level, and insulin resistance in mice in the FF-S group. Histological analysis suggested high degree of steatosis and lobular inflammation and high NAFLD activity score in FF-S and FF-A groups. Results of RT-qPCR showed increased mRNA level of genes encoding fibrosis and inflammatory markers and lipid modulators such as PPAR-γ. Results of the western blot analysis also showed prominent expression of inflammatory and metabolic markers. These results indicate that the short-term administration of the FF diet supplemented with fructose induced the development of NASH and metabolic syndrome.

Moreover, we found that mice in the NC-A group showed higher ALT level, wider necrotic area, and higher degree of DNA fragmentation compared with mice in the FF-A group. These results indicate that APAP-induced ALI was less severe in mice with NAFLD and are consistent with those of several previous studies [14, 15]. Subsequent analyses were performed to determine the mechanisms underlying the effects of NAFLD on APAP-induced ALI.

APAP undergoes CYP2E1-catalyzed oxidation after glucuronidation and sulfation, resulting in NAPQI production [4]. The mRNA levels of genes encoding UGT1A1 and UGT1A9 were not significantly different between NC-A and FF-A groups. However, hepatic CYP2E1 level increased in mice fed the FF diet, which may cause NAPQI formation and APAP-induced ALI. Therefore, this metabolic mechanism cannot be the cause of ALI results of the present study, which showed severe APAP-induced ALI in mice in NC-S group.

NAPQI is detoxified by hepatic GSH. However, excessive amount of NAPQI cannot be detoxified, resulting in liver damage, mitochondrial dysfunction, and necrosis [4]. *Trib3* mRNA level significantly increased in mice in the NC-S group, indicating increased GSH depletion. This may be attributed to the oxidative stress and the multiple molecules involved in the response to oxidative stress [22].

NRF2, a transcription factor, exerts protective effects against hepatotoxicity induced by different agents [23]. Moreover, its activation is involved in GSH production [24]. Under normal condition, NRF2 remains bound to KEAP1 in the cytosol. However, in the presence of oxidative stress, NRF2 detaches from KEAP1 and enters the nucleus to regulate the transcription of genes containing antioxidant response element in their promoter regions [25]. NRF2 regulates genes including *Gclc*, *Gclm*, *Ho-1*, *Gstm1*, *Gpx2*, and *Nqo-1*. The present study showed that the mRNA levels of *Gpx2* and *Gstm1* were significantly higher in mice in the FF-A group than in mice in the NC-A group (RR = 1.90 and 1.51, respectively), indicating that NRF2 activation protected mice in the FF-A group from APAP-induced hepatotoxicity. It is known that several molecules such as p62 and PPAR-γ activate NRF2 [24–27].

Expression of PPAR-γ, which exerts anti-inflammatory effects [28, 29], increased in mice fed the FF diet. This result is consistent with the results of previous studies that showed mild PPAR-γ expression in the normal liver but increased PPAR-γ expression in the livers of obese patients or patients with NAFLD [30, 31]. Several studies have shown that PPAR-γ activation alleviates APAP-induced ALI [32, 33]. These protective effects of PPAR-γ activation against APAP-induced ALI are associated with the elevation of antioxidant enzymes such as GPx and GST that is consistent with our results of mRNA levels. In addition, these observations were also explained by the changes in the expression levels of hepatic nicotinamide adenine dinucleotide phosphate oxidase, ERK, and NF-κB. In the present study, we also detected low levels of p-ERK, NF-κB, p-MKK4, and p-JNK in mice in the FF-A group, which showed high PPAR-γ level in response to APAP-induced oxidative stress. The anti-inflammatory effect of PPAR-γ can also be attributed to the IFN-γ downregulation as PPAR-γ inhibits c-Jun-mediated activation of the IFN-γ promoter [34, 35]. IFN-γ plays a major role in the immune response in APAP-induced ALI [36, 37]. These findings indicate that PPAR-γ decreased the immune response in mice in the FF-A group.

The mRNA level of the gene encoding cyclin D1, which promotes G1-S phase cell cycle progression, increased in mice in the FF-S and FF-A groups. Moreover, the mRNA level of the gene encoding p21, a potent cyclin-dependent kinase (CDK) inhibitor, was lower in mice in the FF-A group than in mice in the NC-A group. P21 overexpression inhibits CDK2 activity, thus promoting the entry of cells into the G0/quiescent phase of the cell cycle [38]. Therefore, increased cyclin D1 and decreased p21 expression in the FF-A group may increase hepatocyte proliferation, which may alleviate APAP-induced ALI by increasing the repair capacity of the liver [24].

Differences between the results of the present study and those of other studies [11, 12, 16] that showed severe APAP-induced ALI in mice with NAFLD may be attributed to the method of establishing NAFLD and the amount of APAP injected. Many studies [11, 12] performed genetic manipulation for establishing NAFLD mice. In these cases, the function of the gene is complex, hence it might affect mechanisms of APAP induced ALI. Therefore, it is difficult to specifically dissect the contribution of NAFLD on APAP induced ALI. In the several studies carried out based on using diet, different kinds of diet such as high fat diet [14, 15] and a diet deficient in methionine and choline [16] were provided. Moreover, fructose supplementation of the FF diet in the present study may have affected the APAP sensitivity by modulating the expression of PPAR-γ [39]. Most studies assessing APAP-induced ALI have used APAP doses of more than 300 mg/kg [11, 12, 14, 16, 40]. This dose is equivalent to a lethal dose compared with the current maximum recommended dosage [4 g/day in adults and 65–75 mg/(kg·day) in children] of APAP [4], and therefore, inappropriate to compare APAP-overdose susceptibility between the control and NAFLD groups. A low dose of APAP proven to cause ALI [41] was administered to compare the susceptibility to APAP-induced ALI in the present study.

In summary, PPAR-γ overexpression during oxidative stress associated with APAP-induced ALI exerts anti-inflammatory effects, activates anti-inflammatory molecules such as NRF2, and inhibits pro-inflammatory molecules such as NF-κB and IFN- γ in mice with NAFLD. Paradoxically, APAP-induced severe oxidative stress along with NAFLD-associated pro-inflammatory state aggressively activates anti-oxidant cascades such as PPAR-γ and NRF2 that alleviates liver damage. These effects decrease the APAP overdose-induced liver injury in mice with NAFLD. Moreover, activation of cell proliferation may help in alleviating ALI rapidly.

However, the present study has some limitations. First, we examined the manifestations of APAP-induced ALI only at a specific time point. Therefore, we could not determine the time-dependent changes in the levels of aforementioned molecules. However, several studies [42, 43] have shown that changes in the levels of some molecules are maximized at 6 h after APAP injection, that is consistent with the early effect of NAFLD against APAP-induced ALI. Second, we could not determine the levels of APAP metabolites because of the limited availability of facilities. Because the accurate levels of APAP glucuronide, APAP sulfate, and NAPQI could not be determined, NAFLD-induced alterations in APAP-induced ALI were indirectly estimated by determining the levels of enzymes involved in each step of APAP metabolism.

Despite these limitations, the present study is the first to use FF diet-induced NAFLD model, which mimics the pathogenesis of human NAFLD, in APAP associated studies. Moreover, this study tried to identify molecules associated with metabolic pathway of APAP as well as proinflammatory and anti-oxidant pathway.

## Conclusions

The results of the present study suggest that FF diet-induced NAFLD exerts early inhibitory effects on APAP-induced ALI. The mechanism is involved by several anti-oxidant molecules such as PPAR-γ and NRF2 in response to severe oxidative stress. However, further studies are need to validate whether NAFLD exerts similar effects on longer duration and other ALI types.

## Additional file

Additional file 1: Table S1. Compositions of the NC and FF diets. FF composed of more fat, cholesterol, carbohydrate and high fructose than NC. NC, normal chow; FF, fast food. **Table S2.** Sequences of sense and antisense primers used in the present study. (DOCX 14 kb)

## Abbreviations

ALI: Acute liver injury
ALT: Alanine aminotransferase
APAP: Acetaminophen
ARE: Antioxidant response element
CDK: Cyclin-dependent kinase
CYP2E1: Cytochrome P450 2E1
dUTP: Nick-end labeling
ERK: Extracellular signal-regulated kinase
FF: Fast food
FF-S: Fast food diet mice followed by saline injection
GSH: Glutathione
HOMA-IR: Homeostasis model assessment of insulin resistance
NAFLD: Non-alcoholic fatty liver disease
NAPQI: *N*-acetyl-*p*-benzoquinone imine
NASH: Non-alcoholic steatohepatitis
NC: Normal chow
NC-S: Normal chow diet mice followed by saline injection
NF-κB: Nuclear factor kappa B
Nrf2: Nuclear factor erythroid 2-related factor 2
p-JNK: Phosphorylated c-Jun N-terminal kinases
p-MKK4: Phosphorylated map kinase kinase 4
PPAR-γ: Peroxisome proliferator-activated receptor gamma
ROS: Reactive oxygen species
RR: Relative ratio
RT-qPCR: Real-time quantitative polymerase chain reaction
SD: Standard deviation
SE: Standard error
TUNEL: Terminal deoxynucleotidyl transferase-mediated
UGT: Uridinediphosphate-glucuronosyltransferase
