# Supplementary material for: Fast food diet-induced non-alcoholic fatty liver disease exerts early protective effect against acetaminophen intoxication in mice
Source: BMC Gastroenterol. 2017 Nov 28;17:124. doi: 10.1186/s12876-017-0680-z (PMC5704433; doi:10.1186/s12876-017-0680-z)
Supplement: Additional file 1: Table S1. — Compositions of the NC and FF diets. FF composed of more fat, cholesterol, carbohydrate and high fructose than NC. NC, normal chow; FF, fast food. Table S2. Sequences of sense and antisense primers used in the present study. (DOCX 14 kb) [file 12876_2017_680_MOESM1_ESM.docx]

Supplementary Table 1. Compositions of the NC and FF diets.

| Composition | Diet | |
| --- | --- | --- |
|  | NC | FF |
| Protein, %wt | 22.6 | 20.0 |
| Fat, %wt | 5.0 | 21.0 |
| Cholesterol, %wt | 0 | 0.21 |
| Carbohydrates, %wt | 39.7 | 50.0 |
| Fiber, %wt | 4.5 | 5.0 |
| HFCS in drinking water, g/l | 0 | 25.0 |

NC, normal chow; FF, fast food.

Supplementary Table 2. Sequences of sense and antisense primers used in the present study.

| Gene | Direction | Primers |
| --- | --- | --- |
| *L32* | Forward | 5ʹ-ggcctctggtgaagcccaagatcg-3ʹ |
|  | Reverse | 5ʹ-cctctgggtttccgccagtttcgc-3ʹ |
| *Col1a1* | Forward | 5ʹ-caccctcaagagcctgagtc-3ʹ |
|  | Reverse | 5ʹ-gttcgggctgatgtaccagt-3ʹ |
| *Tgf-β* | Forward | 5ʹ-tctctgtggagctgaagcaa-3ʹ |
|  | Reverse | 5ʹ-tgagtggctgtcttttgacg-3ʹ |
| *Tnf-α* | Forward | 5’- cccacaccgtcagccgattt - 3’ |
|  | Reverse | 5ʹ-gcttaagtacttgggcagattgacc-3 ʹ |
| *Il-6* | Forward | 5ʹ-agttgccttcttgggactga-3ʹ |
|  | Reverse | 5ʹ-tttccacgatttcccagaga-3ʹ |
| *Il-1β* | Forward | 5ʹ-tgaaatgccaccttttgaca-3ʹ |
|  | Reverse | 5ʹ-cttctccacagccacaatga-3ʹ |
| Resistin | Forward | 5ʹ-cagaaggcacagcagtcttg-3ʹ |
|  | Reverse | 5ʹ-gaccggaggacatcagacat-3ʹ |
| *Ppar-γ1* | Forward | 5ʹ-gagtgtgacgacaagatttg-3ʹ |
|  | Reverse | 5ʹ-ggtgggccagaatggcatct-3ʹ |
| *Ppar-γ2* | Forward | 5ʹ-tctgggagattctcctgttga-3ʹ |
|  | Reverse | 5ʹ-ggtgggccagaatggcatct-3ʹ |
| *Ugt1a1* | Forward | 5ʹ-cctatgggtcacttgccact-3ʹ |
|  | Reverse | 5ʹ- atggctttcttctccggaat-3ʹ |
| *Ugt1a9* | Forward | 5ʹ-acaccggaactagaccatcg-3ʹ |
|  | Reverse | 5ʹ-ataccatgggagccagagtg-3ʹ |
| *Cyp2e1* | Forward | 5ʹ-atgtcatccccaagggtaca-3ʹ |
|  | Reverse | 5ʹ-aggccttctccaacacacac-3ʹ |
| *Trib3* | Forward | 5ʹ-gatgccaagtgtccagtcct-3ʹ |
|  | Reverse | 5ʹ-cttgctctcgttccaaaagg-3ʹ |
| *p21* | Forward | 5ʹ-cggtggaactttgacttcgt-3ʹ |
|  | Reverse | 5ʹ- cagggcagaggaagtactgg-3ʹ |
| Cyclin D1 | Forward | 5ʹ-gcgtaccctgacaccaatct-3ʹ |
|  | Reverse | 5ʹ-ctcttcgcacttctgctcct-3ʹ |
| *Gpx2* | Forward | 5ʹ-ggcttacattgccaagtcgt-3ʹ |
|  | Reverse | 5ʹ-ccgggtagttgttcctcaga-3ʹ |
| *Gstm1* | Forward | 5ʹ-agaaccaggtcatggacacc-3ʹ |
|  | Reverse | 5ʹ-acttgggctcaaacatacgg-3ʹ |
